# Supplementary figures and images for: The combination of zalfermin and semaglutide has additive therapeutic effects in a diet-induced obese and biopsy-confirmed mouse model of MASH
Source: PLoS One. 2025 Oct 28;20(10):e0331665. doi: 10.1371/journal.pone.0331665 (PMC12561900; doi:10.1371/journal.pone.0331665)

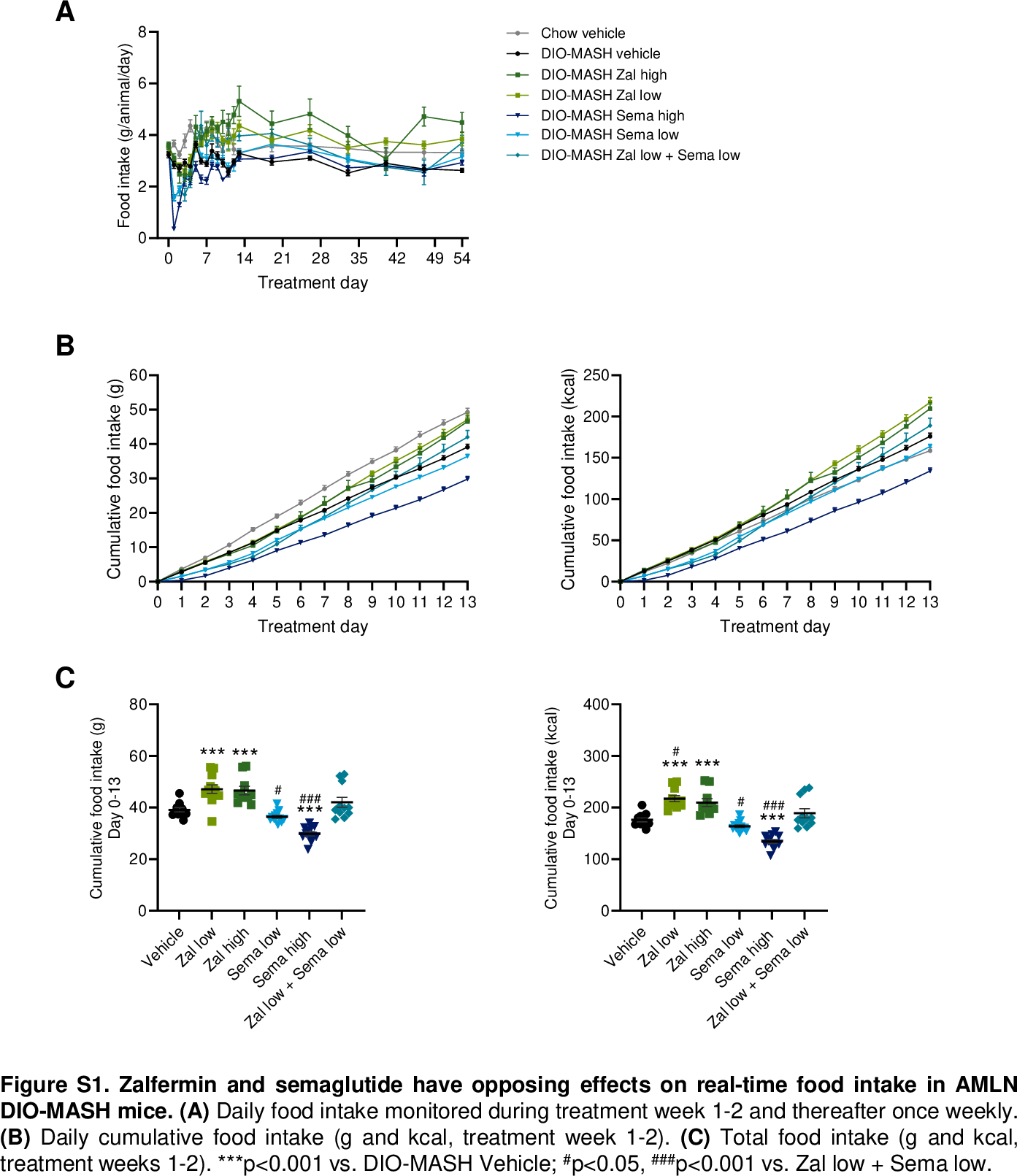

Supplement: S1 Fig — (A) Daily food intake monitored during treatment week 1–2 and thereafter once weekly. (B) Daily cumulative food intake (g and kcal, treatment week 1–2). (C) Total food intake (g and kcal, treatment week 1–2). ***p < 0.001 vs. DIO-MASH vehicle; #p < 0.05, ###p < 0.001 vs. Zal low + Sema low. (TIF) [file pone.0331665.s001.tif]

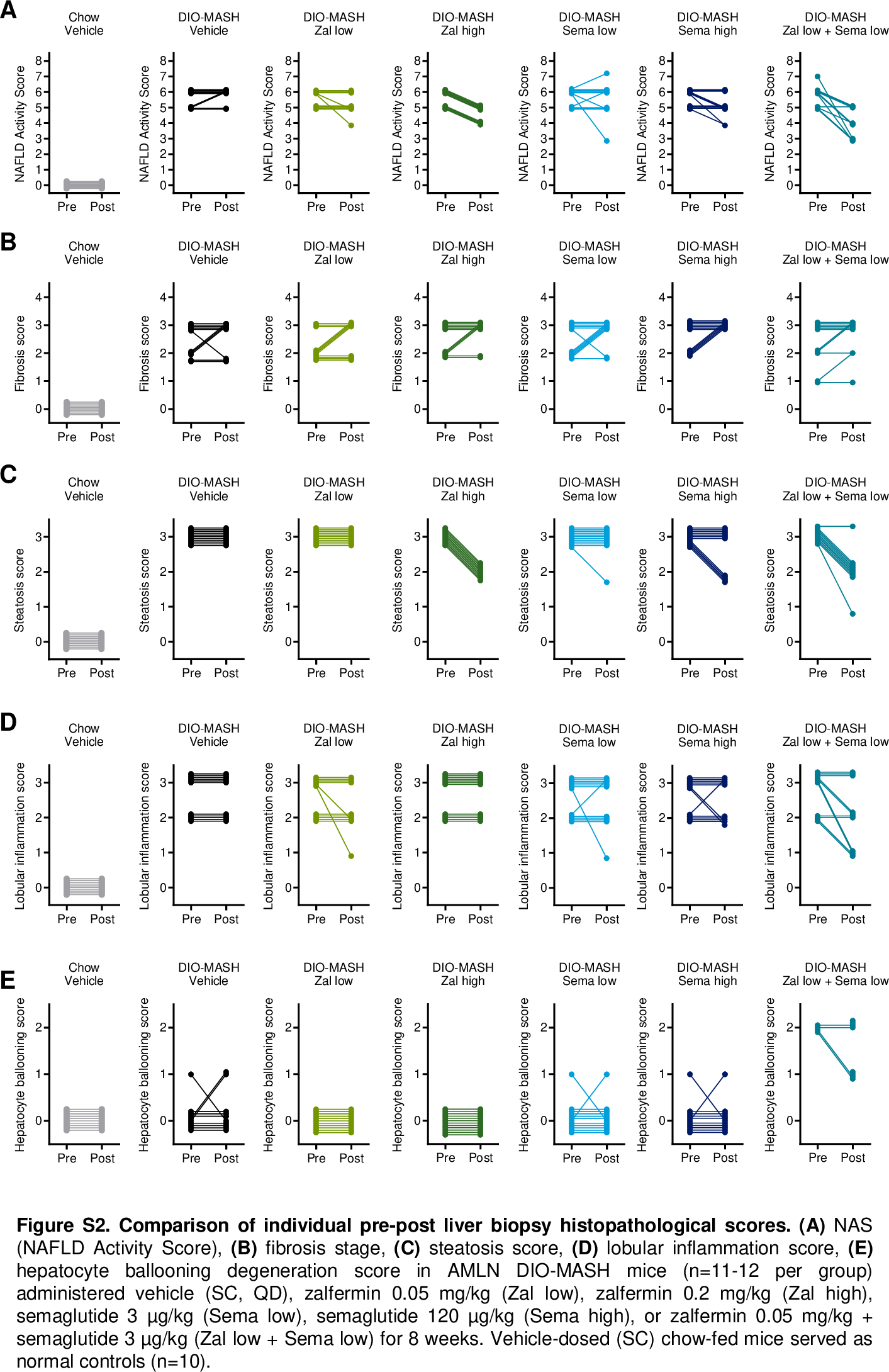

Supplement: S2 Fig — (A) NAS (NAFLD Activity Score), (B) fibrosis stage, (C) steatosis score, (D) lobular inflammation score, (E) hepatocyte ballooning degeneration score in AMLN DIO-MASH mice (n = 11–12 per group) administered vehicle (SC, QD), zalfermin 0.05 mg/kg (Zal low), zalfermin 0.2 mg/kg (Zal high), semaglutide 3 μg/kg (Sema low), semaglutide 120 μg/kg (Sema high), or zalfermin 0.05 mg/kg + semaglutide 3 μg/kg (Zal low + Sema low) for 8 weeks. Vehicle-dosed (SC) chow-fed mice served as normal controls (n = 10). (TIF) [file pone.0331665.s002.tif]

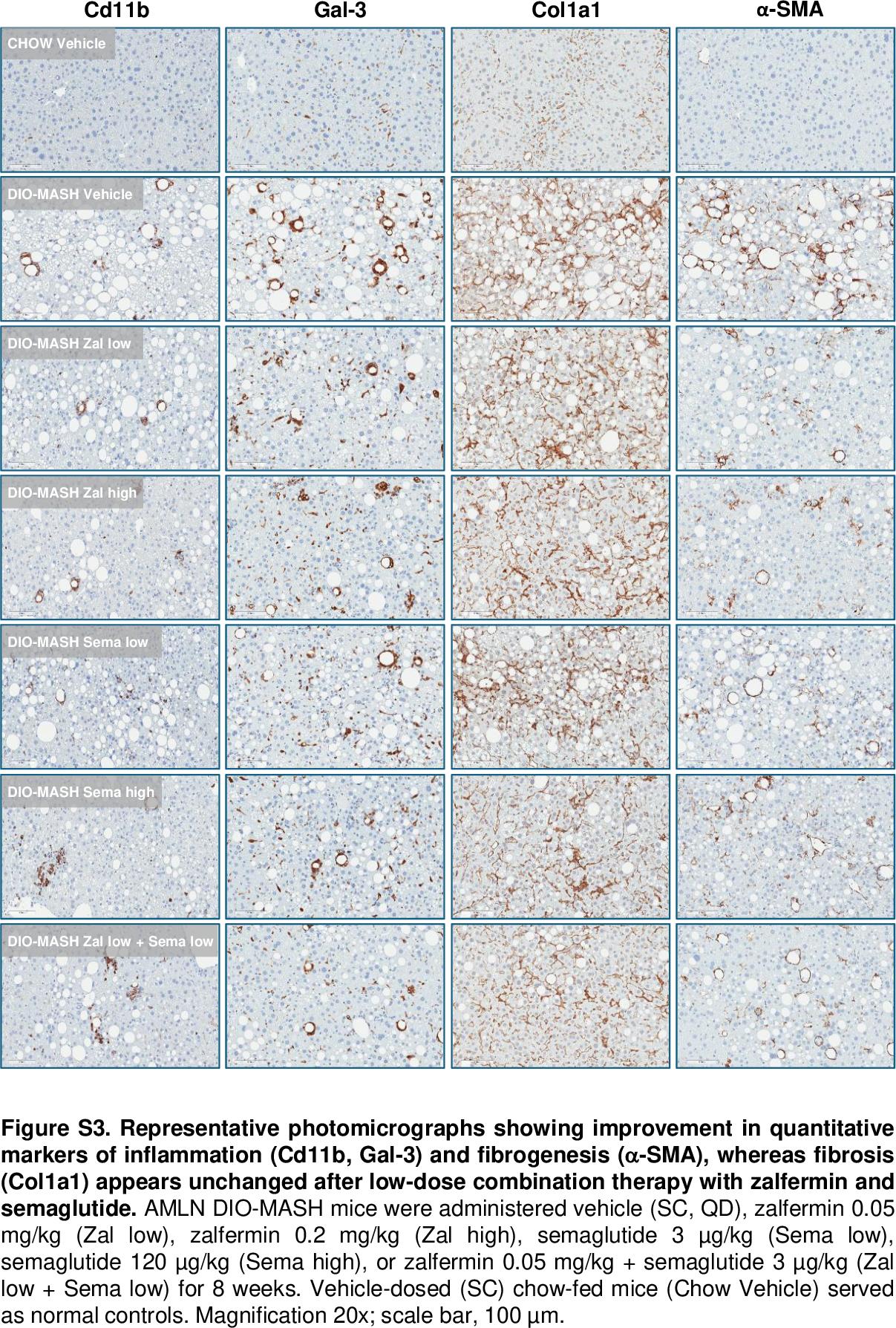

Supplement: S3 Fig — AMLN DIO-MASH mice were administered vehicle (SC, QD), zalfermin 0.05 mg/kg (Zal low), zalfermin 0.2 mg/kg (Zal high), semaglutide 3 μg/kg (Sema low), semaglutide 120 μg/kg (Sema high), or zalfermin 0.05 mg/kg + semaglutide 3 μg/kg (Zal low + Sema low) for 8 weeks. Vehicle-dosed (SC) chow-fed mice (Chow Vehicle) served as normal controls. Magnification 20 ×; scale bar, 100 μm. (TIF) [file pone.0331665.s003.tif]

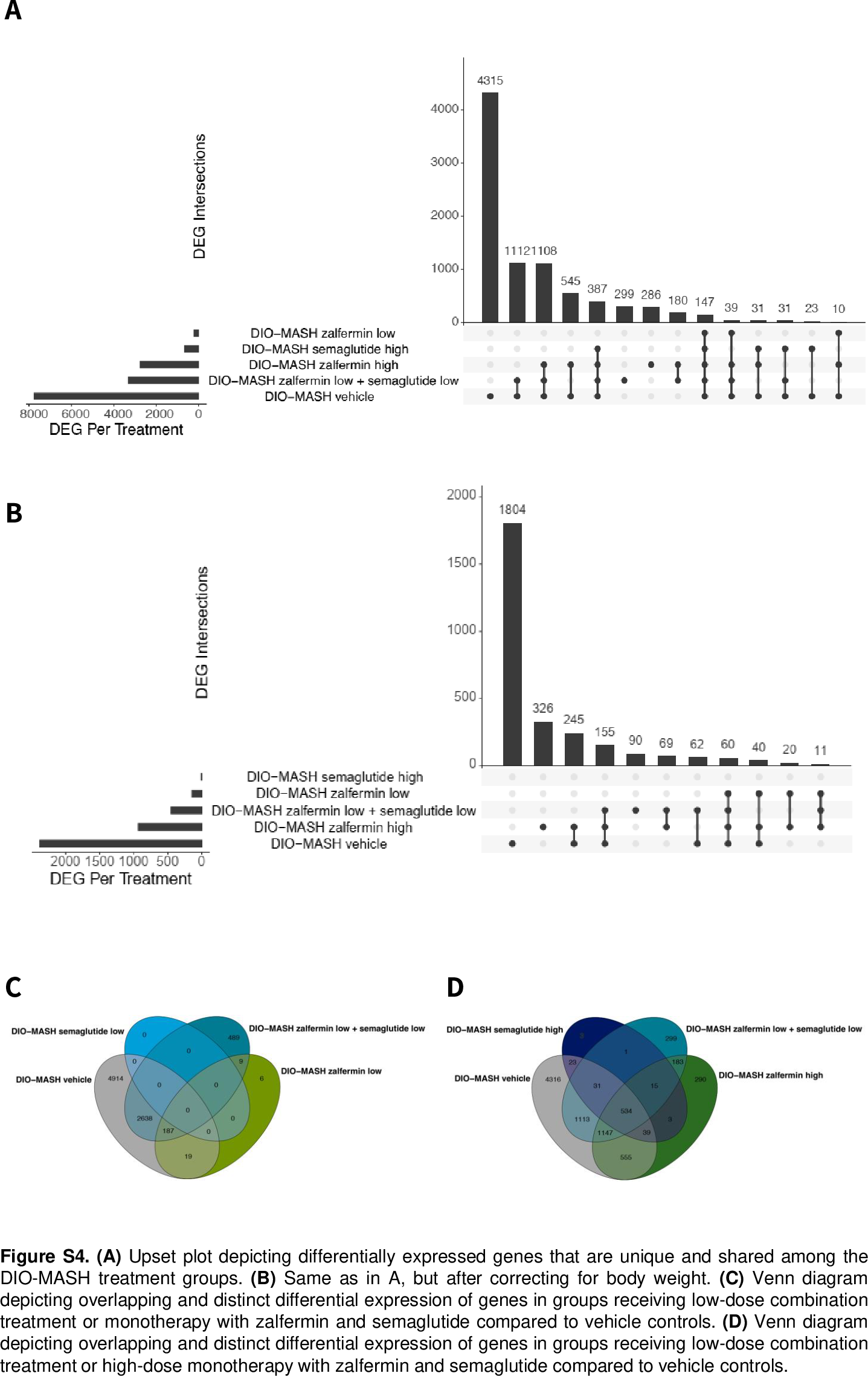

Supplement: S4 Fig — (A) Upset plot depicting DEGs that are unique and shared among the DIO-MASH treatment groups. (B) As A, after correcting for body weight. (C) Venn diagram depicting overlapping and distinct differential expression of genes in groups receiving low-dose combination treatment or monotherapy with zalfermin and semaglutide compared with vehicle controls. (D) Venn diagram depicting overlapping and distinct differential expression of genes in groups receiving low-dose combination treatment or high-dose monotherapy with zalfermin and semaglutide compared with vehicle controls. (TIF) [file pone.0331665.s004.tif]

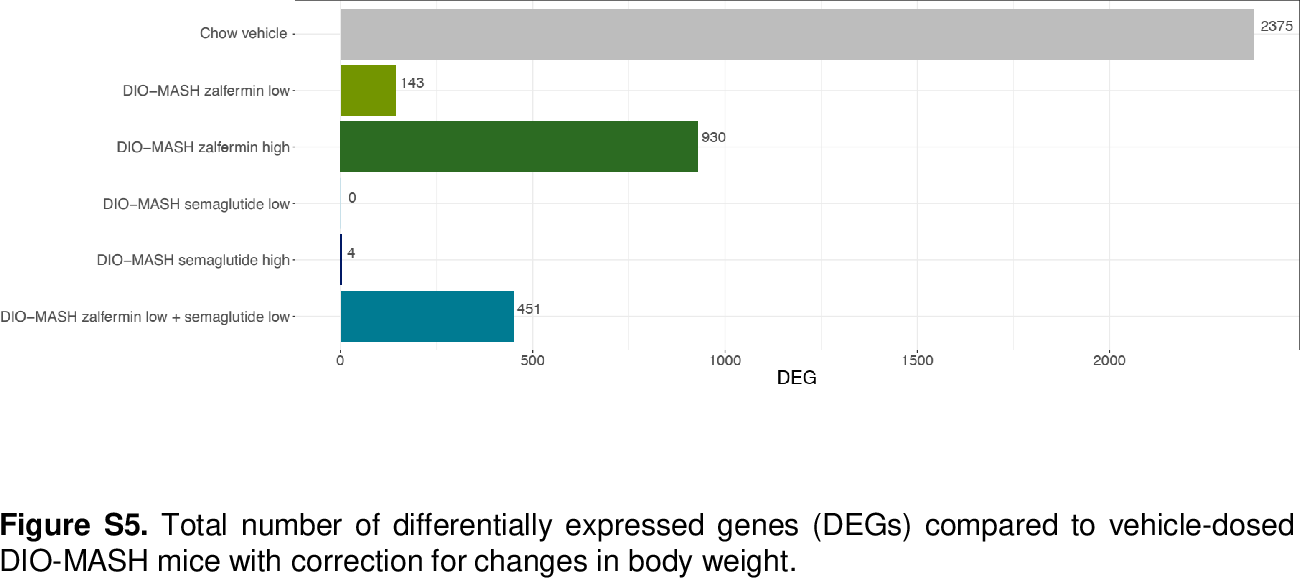

Supplement: S5 Fig — (TIF) [file pone.0331665.s005.tif]
